# Complete mitochondrial genomes of the Arctic charr *Salvelinus alpinus alpinus* Linnaeus (Salmoniformes: Salmonidae)

**DOI:** 10.1080/23802359.2020.1791015

**Published:** 2020-07-21

**Authors:** Alla G. Oleinik, Lubov A. Skurikhina, Andrey D. Kukhlevsky, Alexander A. Semenchenko

**Affiliations:** a A.V. Zhirmunsky National Scientific Center of Marine Biology, Far Eastern Branch, Russian Academy of Sciences, Vladivostok, Russia; b Far Eastern Federal University, Vladivostok, Russia

**Keywords:** Charr genus Salvelinus, Arctic charr, *Salvelinus alpinus*, mtDNA, mitogenome

## Abstract

The complete mitochondrial genomes were sequenced in three individuals of Arctic charr *Salvelinus alpinus alpinus*. The genome sequences were 16,655 and 16,657 bp in length, and the gene arrangement, composition, and size are very similar to the charr genomes published previously. The difference between the three genomes studied is low, 0.09%. The level of sequence divergence between *S. alpinus alpinus* and *S. alpinus oquassa* inferred from the complete mitochondrial genomes is relatively low (0.36%), indicating recent divergence of the taxa and/or potential historical hybridization between different lineages upon secondary contact.

The Arctic charr *Salvelinus alpinus* are salmonid fish distributed throughout the northern circumpolar region and are distinguished by a variability in morphology, coloration, and ecology (Reist et al. 2013). There are several subspecies that were commonly proposed (*S. alpinus alpinus*, *S. alpinus erythrinus*, and *S. alpinus oquassa*) based on their zoogeographic patterns and morphological characteristics. The complete mitochondrial genomes (mitogenomes) of all subspecies of *S. alpinus* have not been established and analyzed in details, and full extent of the evolutionary radiation within the Arctic charr remains unresolved (Oleinik et al. 2015, and references therein). In this study, we examined the mitogenomes of *S. alpinus alpinus* and compared them with charrs from East Asia to North America to assess the phylogeographic patterns of Arctic charr.

We sequenced and described three mitogenomes of *S. alpinus alpinus* from Lake Sitasjaure, Scandinavian Peninsula (67°53′N, 17°37′E). The fish specimens are stored in the collection of the Genetics Laboratory, NSCMB FEB RAS, Vladivostok, Russia (www.imb.dvo.ru) with accession numbers ARLS04.001, ARLS04.004, and ARLS04.014. Totally five pairs of primers were used, which were designed based on public sequences available in GenBank for salmonid fishes. Mitogenome of *S. alpinus* (GenBank accession number AF154851) from introgressed brook charr of Lake Alain, northeastern Québec, Canada (Doiron et al. 2002), found also in native Arctic charr from that area was included for comparison. Our previous results (Oleinik, Skurikhina, Kukhlevsky, Bondar 2019) suggest that the mitogenome AF154851 belongs to *S. alpinus oquassa*.

The size of the mitogenomes was 16,655 bp in *S. alpinus alpinus* (MN957796, and MN957797) and 16,657 bp in *S. alpinus alpinus* (MN957795). The genome organization and GC content (45.6%) is consistent with the mitogenomes of charr species (Balakirev et al. 2016; Oleinik, Skurikhina, Kukhlevsky, Semenchenko 2019). We detected 24 single-nucleotide substitutions and one length difference between the sequences *S. alpinus alpinus* (MN957795) and *S. alpinus alpinus* (MN957796, and MN957797). At the same time, there were 73 single nucleotide (in the CR, tRNA, 12S rRNA, 16S rRNA, and protein coding sequences) and one length differences between the *S. alpinus oquassa* and *S. alpinus alpinus* mitogenomes. Protein coding genes had a different degree of variability, but variability of the NADH dehydrogenase subunit genes was the highest (42.6% proportion of all variable sites). The total sequence divergence (*Dxy*) was 0.0009 ± 0.0003.

The comparison of mitogenomes now obtained with 21 mitogenomes of related groups available in GenBank including genera *Salvelinus*, *Parahucho*, and *Salmo* point to the close relationships of *S. alpinus alpinus* to other charr species (Figure 1). *S. alpinus alpinus* mitogenomes studied here showed similar sequence divergence (0.0075 ± 0.0007 on average) from the GenBank mitogenomes of *S. malma malma*, *S. malma kuznetzovi*, and *S. albus*. These values corresponded to the level of interspecific variability in the genus (Oleinik et al. 2015). The total sequence divergence between *S. alpinus alpinus* and *S. alpinus oquassa* is low, 0.0036 ± 0.0004. The differences between two divergent mtDNA lineages show recent divergence and/or potential historical hybridization between different lineages upon secondary contact (Moore et al. 2015).

**Figure 1.**
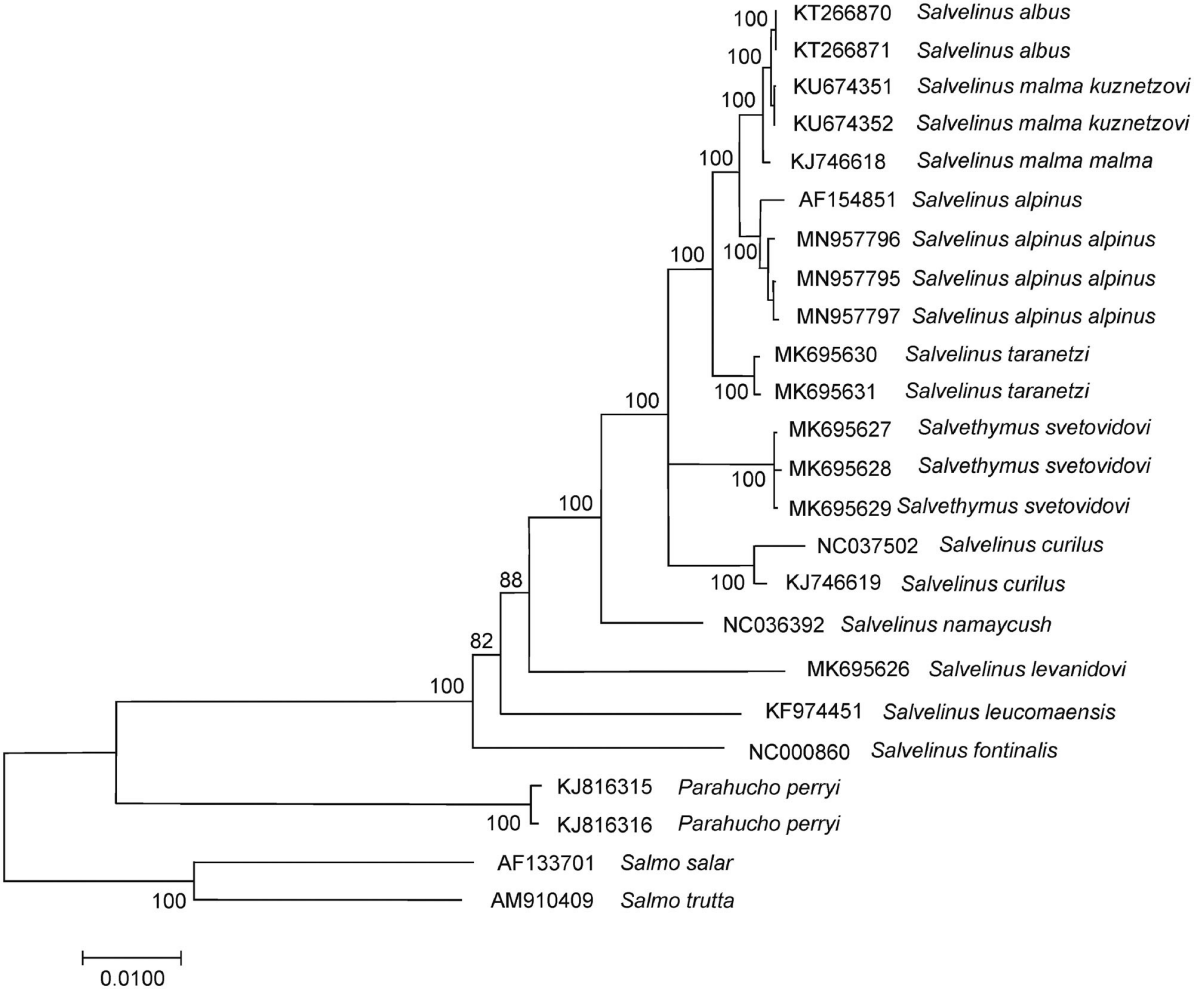
Maximum likelihood (ML) tree constructed on the comparison of complete mitochondrial genome sequences of *Salvelinus alpinus alpinus* and other GenBank representatives of the family Salmonidae. The tree is based on the GTR plus gamma plus invariant sites (GTR + G+I) model of nucleotide substitution. Genbank accession numbers for all sequences are listed in the figure. Numbers at the nodes indicate bootstrap probabilities from 1000 replications (values below 80% are omitted). Phylogenetic analysis was conducted in MEGA X (Kumar et al. 2018).

## Data Availability

The data that support the findings of this study are openly available in the National Center for Biotechnology Information database (NCBI/GenBank) at https://https.ncbi.nlm.nih.gov/, reference numbers MN957795, MN957796, and MN957797.
